# Case report of intracranial tumor with serum MOG-IgG positivity

**DOI:** 10.3389/fimmu.2025.1673927

**Published:** 2025-12-12

**Authors:** Zhuo Min, Lingru Wang, Yulai Kang, Lili Zhang

**Affiliations:** 1 Department of Neurology and Centre for Clinical Neuroscience, Daping Hospital, Army Medical Centre of People’s Liberation Army (PLA), Army Medical University, Chongqing, China; 2 Department of Internal Medicine, No. 93285 Hospital of People’s Liberation Army (PLA), Jilin, China

**Keywords:** MOG-IgG, MOGAD, PET-MRI, coexistence, intracranial tumor

## Abstract

**Objective:**

To deepen understanding of the correlation between intracranial tumor and serum myelin oligodendrocyte glycoprotein immunoglobulin G (MOG-IgG) positivity, emphasizing the importance of avoiding misdiagnosis.

**Methods:**

We report a case involving a 24-year-old Chinese male whose clinical manifestations included slowed responsiveness, dysarthria, and right-sided limb weakness. Beyond serum MOG-IgG positivity, the nature of the central nervous system lesion was further evaluated based on therapeutic response and multimodal imaging.

**Results:**

The patient exhibited serum MOG-IgG positivity with an upward trend in titers. Immunotherapy failed to provide sustained and effective control of disease progression. Integrating the therapeutic response and characteristic imaging changes, a final diagnosis of malignant intracranial tumor was reached. Following diagnosis, he declined further oncologic therapy and died one month later due to a pulmonary infection.

**Conclusion:**

Serum MOG-IgG positivity and an initial response to immunotherapy may be misleading and contribute to diagnostic confusion with inflammatory demyelinating disorders. In atypical or treatment-refractory MOG antibody-associated disease, clinicians should remain vigilant for the possibility of a central nervous system space-occupying lesion.

## Introduction

Myelin oligodendrocyte glycoprotein antibody–associated disease (MOGAD) is defined as a demyelinating disorder of the central nervous system (CNS) linked to myelin oligodendrocyte glycoprotein antibodies (MOG-IgG). The clinical manifestations of MOGAD are highly heterogeneous, encompassing various phenotypes such as optic neuritis, myelitis, acute disseminated encephalomyelitis, brainstem syndromes, and encephalitis. Its diagnosis primarily relies on the detection of MOG-IgG, neuroimaging findings, and the exclusion of other potential causes. Most MOGAD patients have a favorable prognosis, showing substantial recovery following immunotherapy (1).

Although serum MOG-IgG positivity is rarely observed in non-MOGAD conditions, there are limited reports concerning its association with neoplastic diseases, particularly intracranial tumor. The role of MOG-IgG in intracranial tumor lesions remains unclear, and intracranial tumor with serum MOG-IgG positivity are often prone to misdiagnosis or missed diagnosis. This paper reports a case of malignant intracranial tumor in a serum MOG-IgG positive patient. The patient was initially suspected of having MOGAD, however, multiple courses of immunotherapy failed to yield an effective response. Ultimately, based on neuroimaging, the diagnosis was confirmed as a malignant intracranial tumor.

## Case presentation

### March 2024

**Figure 1:**
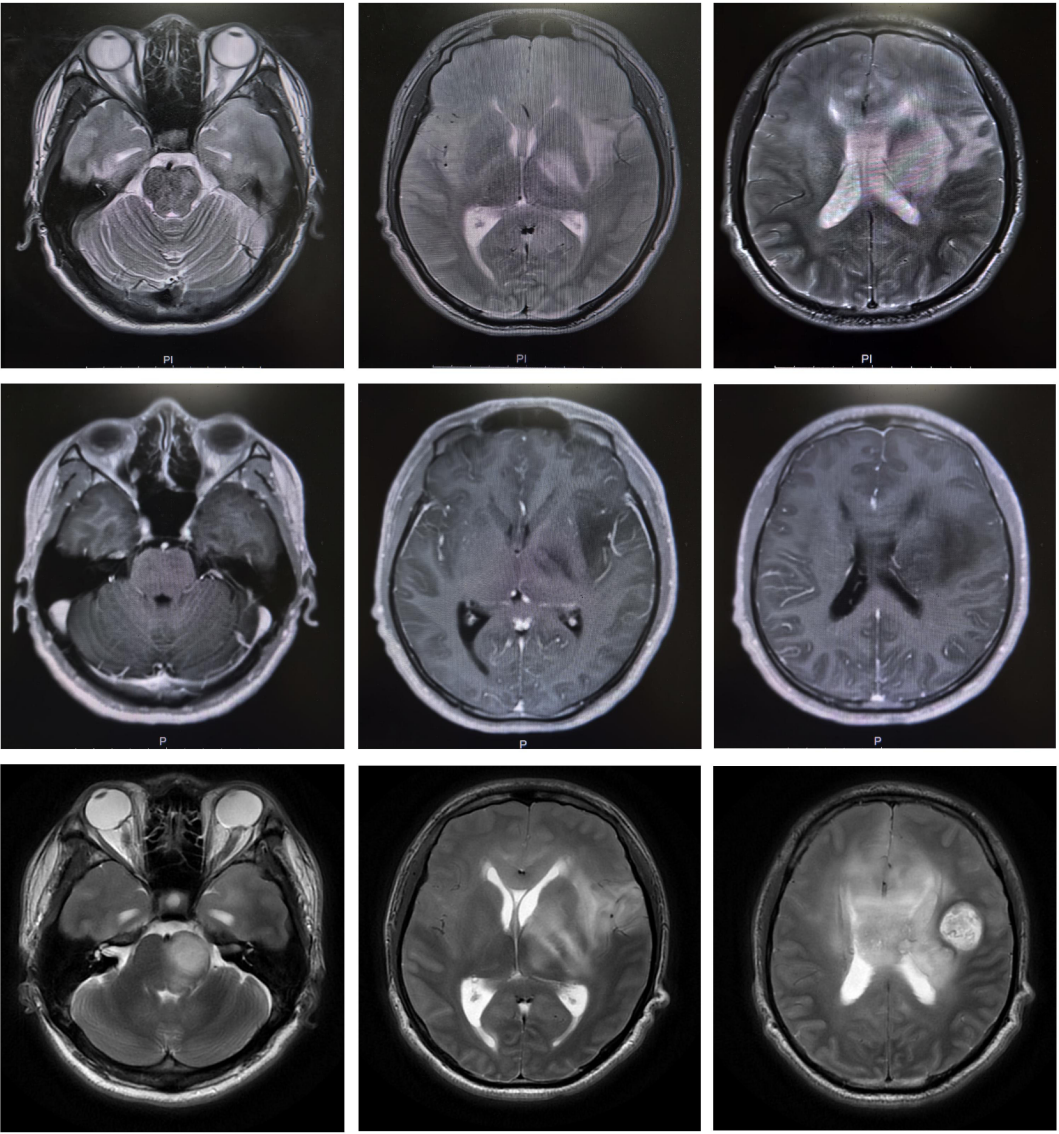
MRI manifestations of intracranial lesions in the patient: Panels **(A1–3)** show axial T2-weighted MRI images obtained at different levels during the patient’s initial onset in March 2024. Patchy hyperintense T2 lesions are observed in the bilateral frontoparietal lobes, the body of the corpus callosum, the left basal ganglia, thalamus, insular-temporal region, and pons. Panels **(B1–3)** present contrast-enhanced sequences; in image B3, linear enhancement can be seen within the lesion adjacent to the left lateral ventricle. Panels **(C1–3)** depict T2-weighted MRI images from a follow-up examination performed at our hospital in August 2024, showing enlargement of the lesion area compared with the prior scan, with a marked mass effect and newly developed nodular long T2 lesions along the inferior margin of the left frontal lobe and the body of the corpus callosum.

A 24-year-old Chinese male presented with sluggish, slurred speech, and weakness in the right limbs. Brain magnetic resonance imaging (MRI) revealed patchy abnormal signals in the bilateral frontoparietal lobes, the body of the corpus callosum, the left basal ganglia region, thalamus, insular-temporal lobe, and pons (**Figures 1A1–3, B1-3**). No abnormal signals were showed in spinal cord MRI. Serum antibody testing demonstrated MOG-IgG positivity (cell-based assay [CBA], titer 1:32), while antibodies against AQP4, GFAP, and MBP were negative. Oligoclonal bands (OCB) of cerebrospinal fluid (CSF) was also negative. MOGAD was considered. Following high-dose corticosteroid pulse therapy, the patient’s symptoms significantly improved.

### May 2024

After a cold, the patient’s symptoms recurred and worsened, with increased right limb weakness and dizziness. Repeat brain MRI at the local hospital showed no significant change compared with the previous MRI. Following intravenous immunoglobulin and high-dose corticosteroid pulse therapy, the symptoms partially improved. After discharge, the patient received oral prednisone combined with mycophenolate mofetil for maintenance therapy, during which the symptoms progressed slowly.

### July 2024

Upon admission to our hospital, neurological examination: Alert but apathetic, with reduced speech and bradyphrenia. Pupils: equal and round bilaterally, approximately 5 mm in diameter. A right central facial palsy was present. Muscle tone in the right limbs: high. Muscle strength: 0/5 in the right upper limb and 3/5 in the right lower limb. Pinprick sensation was diminished on the right. Deep tendon reflexes were hyperactive bilaterally, with positive Hoffmann and Babinski signs and other pyramidal tract signs.

**Figure 2:**
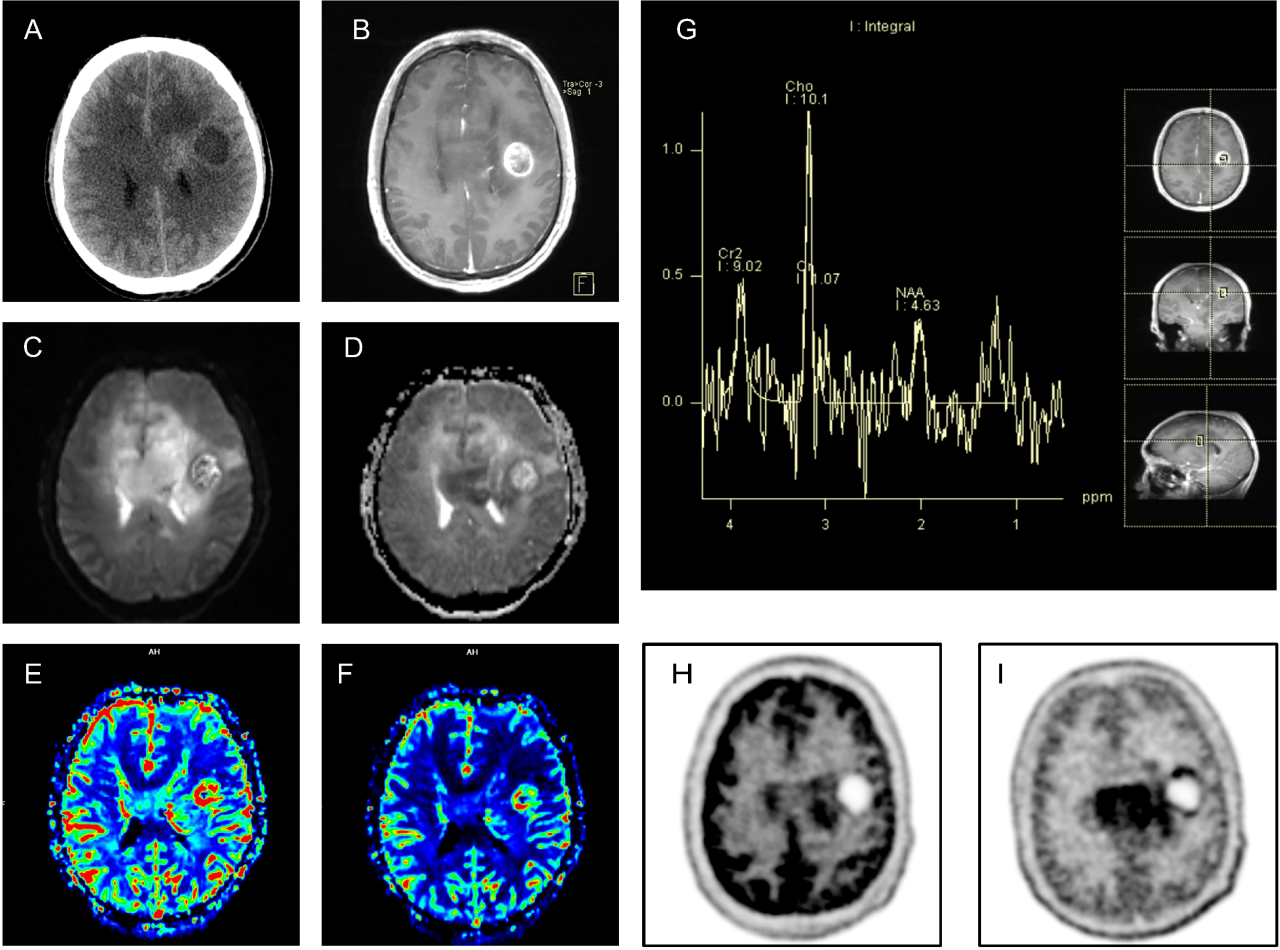
The patient’s cranial imaging demonstrates features consistent with malignant tumor lesions: Panel **(A)**, cranial CT, reveals a pronounced mass effect, midline shift, lateral ventricle compression, and a focal area within the left corpus callosum demonstrating increased density; Panel **(B)**, contrast-enhanced MRI sequence, shows a nodular lesion with complete ring-like enhancement and heterogeneous internal enhancement within the lesion; Panels **(C, D)**, DWI and ADC sequences, demonstrate marked diffusion restriction in the corpus callosum and left periventricular region (hyperintensity on DWI, hypointensity on ADC); Panels **(E, F)**, PWI sequence CBV and CBF maps, reveal increased perfusion in the nodular lesion and left periventricular region; Panel **(G)**, MRS analysis, shows an elevated Cho/NAA ratio in the lesion site, reaching 2.18; Panels **(H, I)**, PET-MRI with radioactive tracers ^18F-FDG and ^11C-MET, indicate significantly increased uptake of both tracers.

Repeat testing showed serum MOG-IgG positivity by CBA (titer 1:320). Head CT (**Figure 2A**) demonstrated compression of the lateral ventricles, rightward deviation of the midline structures, and nodular mass-like lesions involving the left temporal lobe and basal ganglia. Spinal cord MRI revealed no abnormal signal. Brain MRI (**Figures 1C1–3**, **2B–G**) showed: 1. A marked increase in intracranial abnormal signal foci compared with the initial presentation, with new nodular lesions in the left temporal lobe and basal ganglia; internal signal was heterogeneous, and no obvious abnormal signal was detected in either optic nerve; 2. The nodular lesions exhibited heterogeneous ring-like enhancement with aggravated perilesional edema; 3. Susceptibility-weighted imaging indicated susceptibility effects in parts of the lesions; 4. Diffusion-weighted imaging revealed punctate low signal within the nodular lesions; 5. Perfusion-weighted imaging showed hyperperfusion; 6. Magnetic resonance spectroscopy demonstrated Cho/NAA ratios > 2 in some regions. PET-MRI (**Figures 2H, I**) indicated increased FDG and MET uptake.

Based on the imaging findings, a clinical diagnosis of malignant CNS tumor was established, although MOGAD could not be entirely excluded. During hospitalization, in view of the marked increase in the patient’s MOG-IgG titer relative to prior testing, plasma exchange and efgartigimod alfa, among other treatments, were administered. The patient experienced transient improvement, followed by re-deterioration with recurrent visual field deficits and aphasia; repeat brain MRI showed no lesion amelioration (images not shown). The patient and family declined tissue biopsy and oncologic therapy; ultimately, owing to progressive disease, the patient passed away.

## Discussion

According to the 2023 diagnostic criteria for MOGAD (2), this patient had a subacute onset with deficits in cerebral and brainstem function, and multifocal demyelination-like lesions involving supratentorial white matter, juxtacortical regions, deep gray matter, and the pons. At disease onset, MOG-IgG was positive by a fixed CBA, while AQP4-IgG and OCB of CSF were negative, thereby excluding neuromyelitis optica spectrum disorder and multiple sclerosis. The patient’s symptoms improved markedly following corticosteroid therapy. A demyelinating disease of the CNS was considered at an outside hospital, with a high likelihood of MOGAD. The clinical phenotype of MOGAD is age-dependent: in adults, optic neuritis and transverse myelitis are most common (present in > 80% of cases); acute disseminated encephalomyelitis (ADEM) is rare (< 10%); brainstem syndromes account for about 6% and often present with dizziness and cranial neuropathies; and cortical encephalitis comprises about 5% with seizures and headache (3). Our patient presented initially with cognitive impairment and focal neurological deficits without optic nerve or spinal cord involvement and the initial MOG-IgG titer was low, warranting cautious differential diagnosis before confirming MOGAD.

Following the first course of corticosteroids, the disease relapsed and progressed. Repeat high-dose steroids and plasma exchange partially alleviated symptoms, but subsequent immunotherapies failed to control the worsening, amounting to an overall failure of immunotherapy. Pure MOG-IgG positivity alone could not account for these findings.

At onset, intracranial lesions showed minimal mass effect with mild enhancement and a multifocal demyelination-like pattern in the cerebrum and brainstem (**Figures 1A, B**), without distinctive neoplastic features. After admission, follow-up neuroimaging revealed worsening edema and mass effect with compression and deformation of the lateral ventricles, newly appeared nodular lesions with complete ring enhancement, and mildly increased CT attenuation in perinodular areas. At this time, the imaging characteristics shifted toward a neoplastic process. Although serological testing showed significantly elevated MOG-IgG titers and approximately 22% of MOGAD cases may manifest similar tumefactive demyelinating lesions (4), multiparametric advanced MRI provided crucial diagnostic evidence for definitive pathological characterization: on diffusion-weighted imaging, neoplasms typically show progressively increasing diffusion restriction as the disease advances due to higher tumor cell density, tight cellular packing, reduced extracellular space, and lower water content, whereas demyelinating lesions trend oppositely (5); on MRS, neoplasms commonly show marked elevation of the choline peak and reduction of NAA due to tumor cell proliferation and neuronal injury, with a Cho/NAA ratio > 1.72 being more suggestive of neoplasm (6); on perfusion imaging, malignant tumors—especially high-grade gliomas—often exhibit increased CBV and CBF in tumor parenchyma due to neovascular proliferation, whereas rapid growth with central cystic degeneration and necrosis manifests as markedly reduced perfusion (7). Our patient’s imaging showed features strongly indicative of neoplasia (**Figure 2**), and PET demonstrated high tracer uptake, reflecting higher tumor grade. Integrating the clinical and imaging features, the patient was diagnosed with a malignant tumor of the CNS. Unfortunately, brain tissue biopsy was never performed, precluding pathological confirmation of tumor type and stage.

Autoantibodies such as Hu, Yo, and amphiphysin are commonly associated with paraneoplastic syndromes; current evidence suggests the paraneoplastic risk of MOG antibodies is low, and MOGAD exhibits paraneoplastic associations only with certain specific tumors (8). A retrospective study collected 17 cases with tumors within two years of MOGAD onset, indicating potential paraneoplastic associations with teratoma, ovarian tumor, and melanoma, with the strongest association to teratoma, in which MOG protein expression was detectable in tumor tissue biopsy. Among the 17 cases, three were primary CNS tumors (astrocytoma, meningioma, and pituitary macroadenoma), all adjudicated as non-PNS-related (9). Subsequent reports of MOGAD or MOG-IgG positivity with concurrent tumors have similarly not shown a clear paraneoplastic relationship with primary CNS tumors (10). Notably, two case reports by Yasunori Uzura et al. and Meng-Ting Cai et al. described primary CNS lymphoma (PCNSL): both patients initially met MOGAD criteria and were biopsy-proven; after steroid-responsive improvement, they relapsed and worsened within months, and PCNSL was diagnosed by histopathology or flow cytometry. In these cases, MOGAD was not considered a paraneoplastic syndrome but rather a prodromal manifestation of PCNSL (11, 12). Our patient’s pattern of partial steroid responsiveness followed by relapse and worsening within two months mirrors these reports, and the lesion locations, CT attenuation, and MRI perfusion characteristics were likewise similar, making PCNSL as a prodromal process likely. Overall, MOGAD does not appear to have a strong paraneoplastic association with primary CNS tumors; instead, being a prodrome of PCNSL is a notable link between the two.

MOG is a type I membrane-bound glycoprotein specifically expressed on oligodendrocytes and myelin in the CNS; its specific antibody, MOG-IgG, is closely associated with MOGAD, but its precise role in disease initiation and progression remains to be clarified. In contrast to pathogenic AQP4-IgG, evidence for direct complement-dependent pathogenicity of MOG-IgG in MOGAD is insufficient. MOG-IgG is more likely to participate partially in the process of pathogenic activation of MOG-specific T cells (13). In neoplastic and other non-MOGAD conditions, the appearance of MOG-IgG may represent a secondary response that does not exert pathogenic effects in the absence of subsequent pathogenic T-cell activation. In the aforementioned PCNSL prodrome cases, serum MOG-IgG remained at a relatively high level during long-term follow-up after chemotherapy cleared the tumor, yet no MOGAD attacks or progression occurred (11). This suggests that MOG-IgG was not produced by tumor-derived B cells and did not display pathogenicity. We speculate that the lack of pathogenic effects of MOG-IgG may stem from suppression of pathogenic T-cell activity by tumor cells via PD-L1 expression or from elimination of pathogenic T cells by tumor-directed therapies. In our case, it cannot be excluded that MOG-IgG exerted pathogenic effects at the initial stage leading to MOGAD; however, after disease relapse and progression with a markedly higher MOG-IgG titer, targeted clearance of MOG-IgG by plasma exchange and an FcRn antagonist produced no substantive clinical or imaging improvement, indicating that MOG-IgG did not play a significant pathogenic role at that stage.

## Conclusion

This case of an intracranial tumor with MOG-IgG positivity further suggests that the coexistence of MOG-IgG and tumor is not necessarily coincidental, and the pathogenic properties of MOG-IgG may shift over the course of disease. In patients with relapsing MOGAD whose prior treatments are ineffective, misdiagnosis and coexisting disease should be considered, and timely, comprehensive imaging reassessment may help avert poor outcomes. Moreover, when MOGAD is suspected but neoplasm—especially PCNSL—has not been fully excluded, corticosteroid therapy should be used with caution.

## Data Availability

The raw data supporting the conclusions of this article will be made available by the authors, without undue reservation.
